# Tendencia temporal y distribución espacial de la mortalidad por enfermedades tropicales desatendidas en Argentina entre 1991 y 2016

**DOI:** 10.26633/RPSP.2019.67

**Published:** 2019-08-30

**Authors:** Guillermo Macías, Hernán Hernández

**Affiliations:** 1 Universidad Nacional de Mar del Plata y Universidad Nacional de La Matanza Universidad Nacional de Mar del Plata y Universidad Nacional de La Matanza Buenos Aires Argentina Universidad Nacional de Mar del Plata y Universidad Nacional de La Matanza, Buenos Aires, Argentina.; 2 Dirección de Estadísticas e Información de Salud Dirección de Estadísticas e Información de Salud. Secretaría de Gobierno de la Salud. Ministerio de Salud y Desarrollo Social Argentina Dirección de Estadísticas e Información de Salud. Secretaría de Gobierno de la Salud. Ministerio de Salud y Desarrollo Social, Argentina.

**Keywords:** Enfermedades desatendidas, mortalidad, tendencias, epidemiología, modelos logísticos, Argentina, Neglected diseases, mortality, trends, epidemiology, logistic models, Argentina, Doenças negligenciadas, mortalidade, tendências, epidemiologia, modelos logísticos, Argentina

## Abstract

**Objetivo.:**

Conocer la mortalidad por enfermedades tropicales desatendidas (ETD) de Argentina entre 1991 y 2016 y su tendencia temporal general y para las causas más frecuentes por edad y sexo.

**Métodos.:**

Se calcularon las tasas de mortalidad por ETD brutas, específicas por edad y ajustadas por edad de Argentina para el período 1991-2016. Se realizó el análisis de la tendencia temporal mediante modelos de regresión joinpoint.

**Resultados.:**

La mortalidad por ETD afecta principalmente a personas mayores de 50 años y a hombres. La tendencia de las tasas de mortalidad ajustadas por edad por ETD muestran un descenso estadísticamente significativo entre 1991 y 2016, con porcentaje medio de cambio anual (AAPC) para ambos sexos de -3,98 (IC 95%: -4,69; -3,25). Respecto a las enfermedades seleccionadas, la equinococosis muestra un descenso continuo de las tasas entre 1991-2016, mientras que en la enfermedad de Chagas pueden identificarse dos períodos, uno de descenso significativo (1991-2008) y otro no (2008-2016). En cuanto a la lepra, se observa un período de incremento brusco y significativo (1991-1998) seguido por otro período entre 1998-2016 de descenso significativo moderado. Las enfermedades seleccionadas se distribuyen fundamentalmente en el noroeste y noreste argentino, a excepción de la equinococosis.

**Conclusiones.:**

Es significativa la tendencia decreciente de la mortalidad por ETD para ambos sexos. Se pone de relieve que, pese al descenso de los últimos años, la mortalidad por ETD representa un importante problema de salud pública.

## Abstract (en)

**Objective.:**

Determine the mortality from neglected tropical diseases (NTD) in Argentina from 1991 to 2016, their overall temporal trend and the trend for the most frequent causes by age and sex.

**Methods.:**

Argentina’s crude age-specific and age-adjusted mortality from NTDs were calculated for the period 1991-2016. The temporal trend was analyzed using joinpoint regression models.

**Results.:**

Mortality from NTDs is a phenomenon observed primarily in people over 50 and men. The trend in the age-adjusted NTD death rates shows a statistically significant decline between 1991 and 2016, with an average annual percentage change (AAPC) for both sexes of -3.98 (CI 95%: -4.69; -3.25). In terms of the selected diseases, a steady decline in the rates for echinococcus is observed in the period 1991-2016, while two periods can be identified for Chagas, one in which the decline is significant (1991-2008) and another in which it is not (2008-2016). With regard to leprosy, a period with a sharp and significant increase is observed (1991-1998), followed by another period (1998-2016) of significant moderate decline. With the exception of echinococcus, the selected diseases are found basically in northwestern and northeastern Argentina.

**Conclusions.:**

The downward trend in mortality from NTDs is significant for both sexes. It is clear that despite the decline in recent years, mortality from NTDs is an important public health problem.

## RESUMO

**Objetivo.:**

Descrever a mortalidade por doenças tropicais negligenciadas na Argentina entre 1991 e 2016 assim como a tendência temporal geral e as causas mais comuns de mortalidade desagregadas por idade e sexo.

**Métodos.:**

Foi realizado o cálculo das taxas de mortalidade brutas por doenças tropicais negligenciadas, específicas por faixa etária e padronizadas por idade, na Argentina para o período de 1991 a 2016. A tendência temporal foi analisada com o uso de modelos de regressão joinpoint.

**Resultados.:**

A mortalidade por doenças tropicais negligenciadas ocorre sobretudo em indivíduos do sexo masculino acima dos 50 anos. A análise da tendência das taxas de mortalidade por doenças tropicais negligenciadas padronizadas por idade demonstra uma redução estatisticamente significativa entre 1991 e 2016, com variação percentual média anual (AAPC) em ambos os sexos de –3,98 (IC 95% –4,69; –3,25). Com relação a doenças específicas, houve uma redução contínua das taxas de mortalidade por equinococose entre 1991 e 2016; para a doença de Chagas, podem ser identificados dois períodos, um de redução significativa (1991–2008) e outro sem redução (2008–2016) e, para hanseníase, houve um período de aumento súbito significativo (1991–1998) seguido por uma redução moderada significativa entre 1998 e 2016. Estas doenças, à exceção da equinococose, estão principalmente distribuídas nas regiões noroeste e nordeste do país.

**Conclusões.:**

Verifica-se uma tendência de declínio significativo da mortalidade por doenças tropicais negligenciadas em ambos os sexos. Deve-se salientar que, apesar da redução ocorrida nos últimos anos, a mortalidade por doenças tropicais negligenciadas continua sendo um importante problema de saúde pública na Argentina.

Más de 1 000 millones de personas están afectadas en el mundo por enfermedades trasmisibles identificadas como desatendidas (ETD), postergadas u olvidadas. Se calcula que por estas causas mueren alrededor de 500 000 personas al año y las infectadas sufren diversos tipos de discapacidades temporales y permanentes, muchas veces con discriminación y estigmatización por las deformidades y lesiones que producen. A su vez, generan pérdida de trabajo e ingresos del núcleo familiar, lo que empeora drásticamente la situación de los enfermos (1,2).

Las ETD comprenden un grupo de entidades nosológicas que se distinguen por estar directamente relacionadas con malas condiciones de vida (residentes de zonas rurales remotas, barrios marginales, grupos étnicos relegados o zonas de conflicto), con acceso inadecuado a los servicios de salud, la educación, al agua potable y al saneamiento básico, y con ambientes tropicales. Estas características concentran los casos de forma casi exclusiva en poblaciones de bajos recursos de países en desarrollo y ocasionan importantes gastos a sus economías (1–4).

Aunque la mayoría de esas enfermedades tengan tratamiento y sean curables con medicamentos que cuestan menos de un dólar por dosis o son donados a través de la Organización Panamericana de la Salud (OPS), el perfil poblacional de los enfermos repercute poco en la agenda de prioridades de la salud pública, básicamente por su escasa incidencia en las decisiones políticas. Además, la inversión en investigación y desarrollo de nuevos y mejores medicamentos es escasa: sólo 4,4% de los nuevos productos aprobados entre 2000 y 2011 fueron para ETD, la mayoría formulaciones nuevas o combinaciones de drogas ya existentes. La ausencia de estadísticas confiables también contribuye a su reducida visibilidad (1,2,5).

Este grupo de enfermedades incluye el dengue, la rabia, la ceguera por tracoma, la úlcera de Buruli, la treponematosis, la lepra (o enfermedad de Hansen), la enfermedad de Chagas, la leishmaniasis, la cisticercosis, la equinococosis o hidatidosis, la filariasis, la esquistosomiasis y las helmintiasis, entre otras (1). En la Región de las Américas, coexisten 14 de estas enfermedades, entre las cuales destacan la enfermedad de Chagas, fasciolasis, geohelmintiasis, lepra y leishmaniasis (2). En Argentina algunas de estas enfermedades alcanzan cifras endémicas y las de mayor prevalencia son la enfermedad de Chagas, la lepra, la leishmaniasis y la equinococosis (6–8).

En la Región, se estima que unos 6 millones de personas padecen la enfermedad de Chagas (30 000 casos de transmisión vectorial al año y 8 000 de transmisión vertical) (9). Esta enfermedad, que se encuentra principalmente en la parte continental de América Latina (endémica en 21 países), se ha detectado recientemente en los Estados Unidos de América, Canadá, países europeos y algunos del Pacífico Occidental, como consecuencia de las migraciones (10). En 2016, en Argentina se notificaron 2 008 casos de enfermedad de Chagas (2 005 congénitos y 3 vectoriales) (8).

En cuanto a la leishmaniasis, en la Región se diagnostican unos 56 000 casos al año en todas sus formas y se considera endémica en 18 países. Con una epidemiología bastante compleja (por importantes variantes en la transmisión, reservorios, vectores y en las especies del parásito), en 2017 se notificó una incidencia regional de leishmaniasis cutánea y mucosa de 22,51 casos por 100 000 habitantes. Los países con mayor incidencia fueron Surinam y Nicaragua (121 y 140 por 100 000, respectivamente). En Brasil, Bolivia y Paraguay se notificaron 17,7, 29,5 y 3,7 por 100 000, respectivamente, y en Argentina, uno de los valores más bajos de la Región (10,27 por 100 000). En relación con la leishmaniasis visceral, Argentina, Brasil y Paraguay se consideran países con transmisión en expansión. Sus tasas de incidencia en 2017 fueron de 0,02, 1,98, y 0,53 por 100 000, respectivamente. Brasil concentra 90% de los casos de leishmaniasis visceral de la Región (9,11,12). 

La hidatidosis es una enfermedad endémica en Argentina, el sur de Brasil, Chile, Perú y Uruguay. Entre 2009 y 2014, se notificaron casi 5 000 nuevos casos al año en la Región. Las tasas de incidencia pueden ascender a más de 50 por 100 000, y la prevalencia puede alcanzar entre 5 y 10% en algunas zonas de la Argentina y Perú (9,13). En 2016, se registraron 683 casos de hidatidosis en Argentina (8).

En 2014, 81% de los casos nuevos de lepra en el mundo se notificaron en tres países: India (125 785 casos), Brasil (31 064 casos) e Indonesia (17 025 casos). En la Región, en los últimos 5 años, 94% de los nuevos casos detectados se localizaron en Brasil y el resto se distribuyó en 23 países. En Argentina, Bolivia, Brasil, Colombia, Cuba, Ecuador, México, Paraguay, República Dominicana y Venezuela se notifican más de 100 casos nuevos por año (176 casos nuevos en Argentina en 2016) (2,8).

Conocer la distribución y magnitud de la mortalidad y su evolución temporal de estas enfermedades es muy importante para su control. El objetivo de este estudio es conocer la morta­lidad por ETD en Argentina entre 1991 y 2016 y su tendencia temporal general y para las causas más frecuentes por edad y sexo.

## MATERIALES Y MÉTODOS

Se realizó un estudio ecológico longitudinal, tomando como unidades de análisis las provincias argentinas. Se analizaron variables individuales (edad y sexo) y agregadas (provincia de residencia y año de la defunción).

Los datos de mortalidad por ETD como causa básica se obtuvieron del Informe Estadístico de Defunción (IED) elaborado por la Dirección de Estadísticas e Información de Salud (DEIS) del Ministerio de Salud y Desarrollo Social de Argentina. Para el análisis de la mortalidad, se consideraron las defunciones ocurridas a causa de las enfermedades incluidas en las listas de la OMS y la OPS y los códigos que el estudio Global de Carga de Enfermedad clasifica como ETD (14–16). Entre 1991 y 1996, los óbitos se codificaron según la Clasificación Internacional de Enfermedades (CIE-9), y entre 1997 y 2016 se utilizaron los códigos de la CIE-10 (17,18). En el análisis se excluyeron los casos sin especificación de sexo o edad.

Para calcular las tasas brutas de mortalidad se utilizaron poblaciones estimadas a 30 de junio de cada año según sexo y edad publicadas por el Instituto Nacional de Estadísticas y Censos (INDEC) (19,20). Para el conjunto de las ETD, se calcularon tasas brutas, ajustadas por edad y específicas por sexo y edad para el total del período y para cada año. Para las enfermedades seleccionadas (enfermedad de Chagas, leishmaniasis, equinococosis y lepra), se calcularon las tasas brutas y ajustadas por edad y por sexo anuales. La selección de estas enfermedades responde a que son las que presentan mayor carga de mortalidad entre las ETD en Argentina.

Para la descripción y para el ajuste de tasas, se utilizaron grupos de edad quinquenales, porque es lo que habitualmente hace la DEIS y porque, si aumenta la agregación de datos, existe el riesgo de esconder la heterogeneidad de las tasas por edad.

Se calcularon las tasas de mortalidad ajustadas por edad para la enfermedad de Chagas, la leishmaniasis, la equinococosis y la lepra para las provincias argentinas y los decenios 1991-2000 y 2007-2016, a fin de representar y comparar períodos extremos. Estas tasas se representan en mapas, categorizadas en cuartiles o terciles (según corresponda) y utilizando esquemas de color secuenciales (21). Para el ajuste por edad de las tasas de mortalidad se utilizó el método directo (con sus intervalos de confianza de 95% (IC95%) utilizando la población mundial estándar definida por la OMS (22).

Se realizó un análisis de tendencia de la mortalidad mediante regresión segmentada (*joinpoint*) de las tasas ajustadas por edad para el conjunto de las ETD por sexo, y para la enfermedad de Chagas, lepra, leishmaniasis y equinococosis en ambos sexos. Los modelos de regresión de *joinpoint* se componen de algunas fases lineales continuas y permiten describir cambios de tendencia en, por ejemplo, tasas de mortalidad, identificando puntos donde ocurren cambios significativos de la pendiente lineal de la tendencia respecto al tiempo. El análisis comienza probando un modelo sin puntos de inflexión (‘*joinpoints*’), es decir, una línea recta, y luego prueba si uno o más *joinpoints* agregados del modelo dan resultados estadísticamente significativos, mediante la prueba de permutación (23,24).

Como medida resumen de la evolución observada en la mortalidad a lo largo del tiempo, se utilizó el porcentaje medio de cambio anual (AAPC: del inglés *Average Annual Percent Change*), sus IC95% y su grado de significación estadística frente a la hipótesis nula de ausencia de cambio en la pendiente. El AAPC describe el porcentaje del aumento o de la disminución que experimentaron las tasas por unidad de tiempo. Si no se observan puntos de inflexión (cero *joinpoint*), el AAPC indica el cambio en ascenso o descenso para todo el periodo analizado; si se presentan uno o más joinpoint, muestra el cambio para cada segmento de la tendencia (23,24). Se seleccionaron los modelos de *k-joinpoints* que fueron significativos con un error alfa menor de 0,05%, utilizando la prueba de permutación (23).

Para elaborar los mapas, se empleó el programa gvSIG 2.1.0 (25) y para el procesamiento de las bases de datos del IED y el cálculo de las tasas brutas, las tasas ajustadas por edad y los IC95%, el programa R Studio (26). Para el cálculo del AAPC se usó el programa *Joinpoint Regression*, versión 4.2.0.2 de la División de Control de Cáncer y Ciencias de Población del Instituto Nacional del Cáncer de los Estados Unidos de América (27).

Este estudio reúne las características necesarias para prescindir de evaluación por un comité de ética según la Resolución 1480/2011 “Guía para Investigaciones con Seres Humanos” del Ministerio de Salud de la Nación Argentina (28). Los datos provistos por la DEIS están protegidos por la ley de secreto estadístico (ley No. 17622/68) y su decreto reglamentario 3110/70 (29).

## RESULTADOS

Las ETD (aquí se hace referencia a todas las ETD según la lista de códigos de CIE-10) fueron responsables de 15 329 muertes en Argentina entre 1991 y 2016, de las cuales 59,8% (9 175) se produjeron en hombres. El 90,4% de las defunciones se produjo en los grupos de más de 49 años de edad. A medida que aumenta la edad, también lo hicieron las tasas de mortalidad específicas por edad, y la del grupo de 80 y más años fue 5 veces mayor que la del grupo de edad de 50-54 años (cuadro 1). El 92,8% (14 222 óbitos) de las defunciones entre 1991-2016 fueron por la enfermedad de Chagas, seguidas por la equinococosis (3,8%; 582), la lepra (2%; 305) y la leishmaniasis (0,6%; 88) (cuadro 2).

Estas enfermedades seleccionadas produjeron mayor mortalidad en hombres que en mujeres. Excepto para las defunciones por leishmaniasis, para el resto la media de edad de los fallecidos se encuentró por encima de los 60 años y, de forma similar a lo que sucedió para todos los casos, la tasa de mortalidad aumentó con la edad. Así, en mujeres de 80 y más años, la mortalidad por enfermedad de Chagas fue 3,5 veces mayor que entre 60-64 años (10,1 frente a 2,9 x 100 000); en hombres esta relación fue de 2,8 veces (17,4 frente a 6,2 x 100 000). Situaciones semejantes se observan en la equinococosis y la lepra: en la primera, la razón entre las tasas de mortalidad entre grupos de 80 y más años y de 60-64 años fue de 5,5 (0,71 frente a 0,13 x 100 000) y 3,1 (0,37 frente a 0,12 x 100 000), y para la lepra, de 5,9 en hombres (0,65 frente a 0,11 x 100 000) y 4,8 (0,19 frente a 0,04 x 100 000) en mujeres.

**CUADRO 1.** Caracterización de las defunciones por enfermedades tropicales desatendidas (tasas por 100 000 habitantes), Argentina, 1991-2016

Características	Defunciones (No.)	Tasa bruta	Tasa ajustada	(IC 95%)
Sexo				
Ambos sexos	15 329	1,54	1,41	(1,39-1,43)
Mujeres	6 130	1,21	0,99	(0,96-1,01)
Hombres	9 175	1,89	1,94	(1,90-1,98)
Grupo de edad				
0-4	90	-	0,10	(0,08-0,12)
5-9	23	-	0,03	(0,02-0,04)
10-14	23	-	0,03	(0,02-0,04)
15-19	29	-	0,03	(0,02-0,05)
20-24	53	-	0,06	(0,05-0,08)
25-29	87	-	0,12	(0,09-0,14)
30-34	203	-	0,29	(0,25-0,33)
35-39	356	-	0,55	(0,49-0,61)
40-44	601	-	1,02	(0,94-1,11)
45-49	850	-	1,58	(1,48-1,69)
49-54	1 207	-	2,48	(2,34-2,63)
54-59	1 628	-	3,74	(3,56-3,93)
60-64	1 816	-	4,72	(4,50-4,94)
65-69	2 035	-	6,21	(5,94-6,49)
70-74	1 824	-	6,97	(6,65-7,29)
75-79	1 716	-	8,89	(8,48-9,32)
80 y +	2 788	-	13,56	(13,06-14,07)

***Fuente:*** elaboración propia a partir de datos de la DEIS y del INDEC.

**FIGURA 1.**
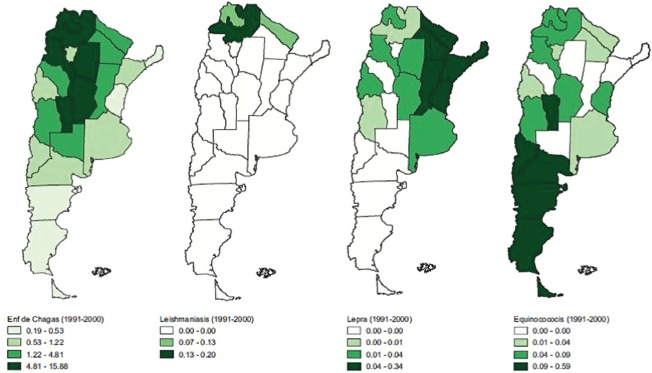
Distribución de las tasas de mortalidad ajustadas por edad de la enfermedad de Chagas, equinococosis, leishmaniasis y lepra, República Argentina, 1991-2000

Las tasas de mortalidad ajustadas por edad de las enfermedades seleccionadas para los decenios 1991-2000 y 2007-2016 a nivel provincial muestran que, a excepción de la equinococosis, la mortalidad fue más frecuente en la región del noroeste y noreste argentino, tanto en el primer como en el segundo decenio. La mortalidad por equinococosis muestra mayores tasas ajustadas por edad en la región patagónica, ubicada en el sur del país. Es importante mencionar que en todas las provincias se produjeron defunciones por enfermedad de Chagas, un fenómeno que no se observó en el resto de las enfermedades seleccionadas (cuadro 2) (figura 1 y 2).

En el análisis de la tendencia temporal de la mortalidad por ETD mediante regresión *joinpoint* se observa que, entre 1991 y 2016, la tendencia temporal de mortalidad por ETD mostró un descenso de las tasas ajustadas por edad para ambos sexos. El AAPC del período para ambos sexos fue de -3,98 (IC 95%: -4,69; -3,25), para hombres de -3,90 (IC95%: -4,75; -3,05) y para mujeres de -3,71 (IC95%: -4,57; -2,84) (figura 3).

El análisis de los AAPC muestra un modelo estadísticamente significativo de descenso de la mortalidad por ETD entre 1991 y 2008 (figura 3). Para el período 2008-2016, el comportamiento de la tendencia de mortalidad por sexo fue heterogéneo. En hombres, se observa una disminución significativa, aunque menor a la del período previo, mientras que en las mujeres se produjo un ascenso de la mortalidad por ETD, aunque este aumento no es estadísticamente significativo (figura 3).

El análisis de la tendencia temporal de la mortalidad por las enfermedades seleccionadas mediante regresión sólo se realizó para la enfermedad de Chagas, la equinococosis y la lepra, no para la leishmaniasis por la presencia tasas de mortalidad con valores 0 en varios periodos.

**CUADRO 2.** Defunciones, tasas de mortalidad bruta y ajustada por edad y sexo (por 100 000 habitantes) por enfermedades tropicales desatendidas (ETD), Argentina, 1991-2016

ETD	CIE-9	CIE-10	Sexo	Edad (media)	Número		Tasa	
						Bruta	Ajustada	(IC95%)
Enfermedad de Chagas	0860/1/2/9	B57	A. sexos Mujeres Varones	62,1 64,7 61,6	14 222 5 689 8 511	1,43 1,12 1,75	1,31 0,92 1,8	(1,29-1,33) (0,89-0,94) (1,76-1,84)
Leishmaniasis	0850-0859	B55	A. sexos Mujeres Varones	52,3 48,9 53,0	88 15 73	0,01 nc 0,02	0,01 nc 0,02	(0,01-0,01) nc (0,01-0,02)
Tripanosomiasis gambiense	0863/4/5	B56	A. sexos Mujeres Varones	70,0 70,0 -	1 1 0	nc nc nc	nc nc nc	nc nc nc
Esquistosomiasis	1200-1209	B65	A. sexos Mujeres Varones	52,8 49,7 62,0	4 3 1	nc nc nc	nc nc nc	nc nc nc
Ascariasis	1270	B77	A. sexos Mujeres Varones	3,1 4,4 1,5	13 7 6	nc nc nc	nc nc nc	nc nc nc
Anquilostomiasis	1260-1269	B76	A. sexos Mujeres Varones	67,0 73,7 47,0	4 3 1	nc nc nc	nc nc nc	nc nc nc
Teniasis	1230/2/3	B68	A. sexos Mujeres Varones	65,0 69,0 61,0	2 1 1	nc nc nc	nc nc nc	nc nc nc
Cisticercosis	1231	B69	A. sexos Mujeres Varones	51,8 47,6 54,0	41 14 27	nc nc 0,01	nc nc 0,01	nc nc (0,00-0,01)
Equinococosis	1220-1229	B67	A. sexos Mujeres Varones	61,5 62,5 60,8	582 269 311	0,06 0,05 0,06	0,05 0,04 0,07	(0,05-0,06) (0,04-0,05) (0,06-0,07)
Filariasis	1250/1/2/4/5/6/9	B74	A. sexos Mujeres Varones	60,4 89,0 53,3	5 1 4	nc nc nc	nc nc nc	nc nc nc
Fascioliasis	1213	B663	A. sexos Mujeres Varones	72,0 72,0 -	1 1 0	nc nc nc	nc nc nc	nc nc nc
Paragonimiasis	1212	B664	A. sexos Mujeres Varones	nc < 1 año -	1 1 0	nc nc nc	nc nc nc	nc nc nc
Lepra	0300-0309 y 1398	A30 y B92	A. sexos Mujeres Varones	67,4 68,0 67,1	305 85 217	0,03 0,02 0,04	0,03 0,01 0,05	(0,02-0,03) (0,01-0,02) (0,04-0,05)
Tracoma	0760/1/9	A71	A. sexos Mujeres Varones	39,2 59,4 22,3	11 5 6	nc nc nc	nc nc nc	nc nc nc
Frambesia	1020-1029	A66	A. sexos Mujeres Varones	68,3 61,0 70,7	5 2 3	nc nc nc	nc nc nc	nc nc nc
Pinta	1030/1/2/3/9	A67	A. sexos Mujeres Varones	84,0 84,0 -	1 1 0	nc nc nc	nc nc nc	nc nc nc
Sífilis no venérea	1040	A65	A. sexos Mujeres Varones	17,0 17,0 -	1 1 0	nc nc nc	nc nc nc	nc nc nc
Rabia	071	A82	A. sexos Mujeres Varones	14,0 - 14,0	3 0 3	nc nc nc	nc nc nc	nc nc nc
Dengue	061	A90	A. sexos Mujeres Varones	60,3 54,8 72,8	20 12 6	nc nc nc	nc nc nc	nc nc nc
Dengue hemorrágico	0654	A91	A. sexos Mujeres Varones	37,4 41,8 20,0	5 4 1	nc nc nc	nc nc nc	nc nc nc

***Fuente:*** elaboración propia a partir de datos de la DEIS y del INDEC. A. sexos: ambos sexos. nc: no calculado porque el número de casos es muy pequeño (menor de 25). Nota: la suma de casos no es igual al total por los casos con sexo no especificado. Las siguientes ETD no presentaron casos en el período estudiado: tricuriasis, oncocercosis, dracontiasis, opistorquiasis, clonorquiasis, ni la infección cutánea por micobacterias (úlcera de Buruli).

***Fuente:*** elaboración propia a partir de datos de la DEIS y del INDEC.

**FIGURA 2.**
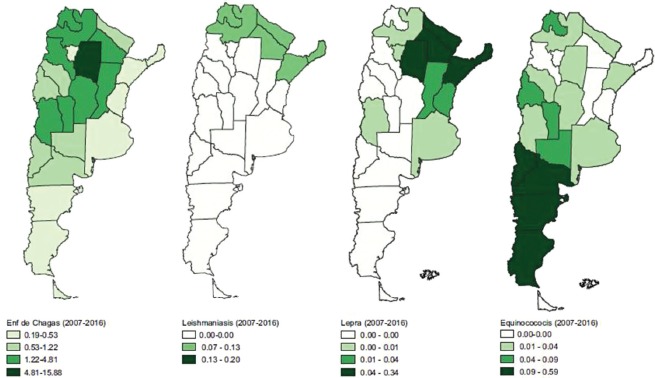
Distribución de las tasas de mortalidad ajustadas por edad de la enfermedad de Chagas, equinococosis, leishmaniasis y lepra, República Argentina, 2007-2016

***Fuente:*** elaboración propia a partir de datos de la DEIS y del INDEC.

**FIGURA 3.**
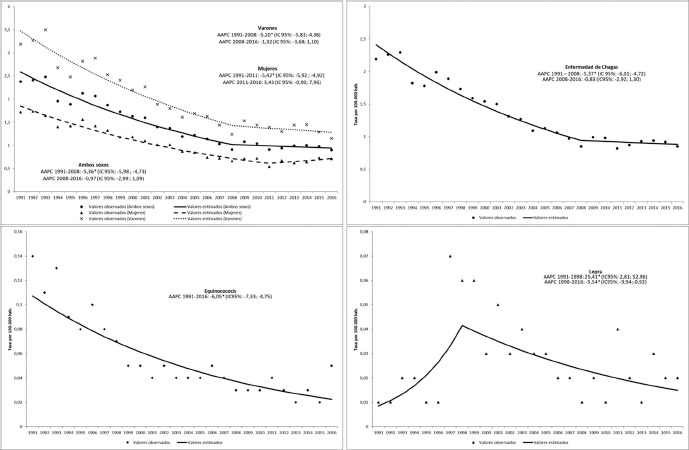
Tendencia de las tasas de mortalidad ajustadas por edad y sexo (por 100 000) por enfermedades tropicales desatendidas seleccionadas (análisis *joinpoint*), Argentina, 1991-2016

***Fuente:*** elaboración propia a partir de datos de la DEIS y del INDEC.

El comportamiento de la tendencia temporal también fue heterogéneo para estas enfermedades. Mientras la equinococosis mostró un descenso continuo de las tasas ajustadas entre 1991-2016 (sin *joinpoint*: AAPC -6,05, IC95%: -7,33; -4,75), para la enfermedad de Chagas pueden identificarse dos períodos, uno de descenso estadísticamente significativo (1991-2008: AAPC -5,37, IC95%: -6,01; -4,72) y otro no (2008-2016: AAPC -0,83, IC95%: -2,92; 1,3). En cuanto a la lepra, se aprecia un período de aumento brusco y significativo entre 1991 y 1998 (AAPC 25,41, IC95%: 2,81; 52,96), seguido de otro período entre 1998 y 2016 de descenso moderado, aunque también significativo (AAPC -5,54, IC95%: -9,94; -0,92) (figura 3).

## DISCUSIÓN

Los resultados de este estudio muestran una fuerte relación entre la mortalidad por ETD y el sexo y la edad. En el período estudiado, más de la mitad de los fallecidos por ETD fueron hombres. Resultados similares han sido descritos por otros autores, que los atribuyen al hecho de que los hombres son menos propensos a buscar tratamiento temprano (30,31). Esta situación también podría deberse a su mayor tiempo de exposición en sus actividades laborales (32). En Argentina, las mujeres refieren peores estados de salud autopercibidos que los hombres, lo que puede influir en la búsqueda de atención (33,34).

La relación incremental entre las tasas específicas de mortalidad por ETD con la edad da cuenta de la cronificación de la historia natural de las ETD (30,31). La distribución espacial de las ETD, a excepción de la equinococosis, muestra las mayores tasas de mortalidad ajustadas por edad, que se observan en el noroeste y el noreste de Argentina. La asociación entre pobreza, urbanización y ETD ha sido analizada por diversos investigadores (1,30,31,35). En las provincias que integran estas regiones se detectan los porcentajes medios de hogares con necesidades básicas insatisfechas (que miden condiciones materiales de vida) de 25% en 2001 y de 17% en 2010, 1,8 veces más altos que el porcentaje nacional (36).

La equinococosis es la única ETD con mayor mortalidad en la región patagónica (sur de la Argentina). Esto podría estar relacionado con el hecho de que en esta zona se concentra la producción ovina del país y a que el autoconsumo tiene valores mayores que la media nacional (1,37,38). Valga mencionar que este tipo de ganado es un huésped intermediario del parásito y fuente de transmisión a humanos (1,37).

Si bien los óbitos por enfermedad de Chagas son los únicos distribuidos en todas las jurisdicciones, las tasas de mortalidad fueron mayores en la región noroeste y noreste, a semejanza de las de otras enfermedades analizadas. Aunque seis jurisdicciones de la Argentina han logrado la interrupción vectorial de la enfermedad, su distribución en todo el territorio nacional se explica por la transmisión vertical y las corrientes migratorias internas (39,40).

El análisis de la tendencia temporal de mortalidad por ETD y para cada uno de los eventos revela un descenso de las tasas ajustadas por edad. Resultados similares se describen para Brasil, con un AAPC de -2,1% (IC95%: -2,8%; -1,3%) en el período 2000-2011 (41). En Argentina, esta reducción puede estar relacionada con la implementación de programas específicos nacionales, como el Programa Nacional de Chagas, el Programa Nacional de Vectores, el Programa Nacional de Enfermedades Zoonóticas, el Programa Nacional de Lepra, etc., y provinciales, así como con las mejoras de las condiciones de vida (36).

No obstante, pese a que muchas de estas enfermedades han cumplido más de 100 años de existencia en el continente (por ej., la enfermedad de Chagas), es importante reflexionar sobre los factores que contribuyen a su persistencia. Zabala analiza la persistencia de la enfermedad de Chagas tras cien años de existencia y el desarrollo de programas y políticas y los desarrollos y descubrimientos en torno a la enfermedad, y hace hincapié en cómo operan las “[…] dimensiones técnicas, biológicas, de conocimiento, profesionales y políticas sobre la tensión que ha atravesado a la enfermedad caracterizada por períodos de reconocimiento y olvido”. El autor sostiene que “[…] la enfermedad de Chagas no ha sido tan olvidada como para desaparecer de la agenda, ni tan recordada como para cortar definitivamente su ciclo de reproducción” (42). Esta reflexión es fácilmente extrapolable al resto de las ETD.

Larrieu revisa críticamente los programas de control de la histoplasmosis para América del Sur y Argentina. En este país, algunos programas datan de la década de los ochenta. El autor describe el fracaso de la erradicación del parásito y lo atribuye a la deficiencia de infraestructura para administrar el fármaco indicado para la desparasitación (43).

Una situación interesante es el notable aumento observado en las tasas de mortalidad por lepra en 1997, precisamente durante el cambio de codificación (de la CIE-9 a la 10ª). Sin embargo, en la CIE-10 no hay nada que sugiera que el cambio de clasificación produjera un aumento, porque, a diferencia de otras enfermedades (por ej., la diabetes), en la lepra no hubo aumento del número de códigos en comparación con la CIE-9. Lo que pudo haber influido es el apoyo de la OPS al programa nacional de lepra ese mismo año (44), ya que en otras publicaciones se indica que dicho programa existía desde 1976, con una estructura vertical de alta calidad pero baja cobertura (45).

El análisis de la mortalidad por ETD como causa básica presenta algunas limitaciones, como la subestimación y el subregistro (30,31,46). Si el médico que registra la defunción selecciona como causa básica las consecuencias de las ETD, la mortalidad se verá subestimada. Un estudio sobre mortalidad por enfermedad de Chagas según causas múltiples realizado en Saõ Paulo (Brasil) entre 1985 y 2006 mostró que cuando ésta es la causa básica de defunción, las causas asociadas son trastornos de la conducción/arritmias (39%) e insuficiencia cardíaca (34%) (46). En cambio, las principales causas básicas cuando la enfermedad de Chagas se registra como causa asociada son la enfermedad isquémica del corazón (22%) y enfermedad cerebrovascular (16,8%). Lo anterior pone de relieve que la mortalidad por enfermedad de Chagas puede estar subregistrada si el médico selecciona las consecuencias cardíacas de la enfermedad en el registro de defunción.

Para concluir, este estudio pone de relieve la magnitud y la distribución de la mortalidad por ETD en Argentina en un período prolongado, con importantes diferenciales en las tasas en las distintas provincias del país. La tendencia en general tuvo valores descendentes hasta el periodo 2008-2011, momento en que se estabilizaron. Todo indica que las ETD aún representan un problema de salud pública muy importante por la población a la que afectan. Si bien la disminución de las tasas de mortalidad por ETD en Argentina en los últimos 25 años ha sido relevante, la detención de esa reducción y los diferenciales regionales indican la necesidad de renovar los esfuerzos en las estrategias de prevención, detección temprana, control y tratamiento de estas enfermedades, debido a las consecuencias aciagas que provocan en grupos humanos más desfavorecidos. Se recomienda la monitorización constante de estas enfermedades y de los programas y proyectos relacionados, la constante difusión de prácticas preventivas entre la población vulnerable e incentivar la investigación y desarrollo de nuevos tratamientos centrados en estas enfermedades.

## Contribuciones de los autores.

Todos los autores han participado en el diseño del estudio original, análisis de los datos, interpretación de los resultados y en la redacción, revisión y aprobación del manuscrito final.

## Financiación.

Los autores declaran no haber recibido ninguna financiación para realizar este estudio.

## Declaración.

Las opiniones expresadas en este manuscrito son responsabilidad de los autores y no reflejan necesariamente los criterios ni la política de la RPSP/PAJPH y/o de la OPS.
